# Healthcare resources for inborn errors of immunity in the Asia-Pacific region

**DOI:** 10.70962/jhi.20250023

**Published:** 2025-05-13

**Authors:** Motoi Yamashita, Yunfei An, Dharmagat Bhattarai, Vicky Wee Eng-Binas, Suravat Homvises, Intan Hakimah Ismail, Mahnaz Jamee, Dae Chul Jeong, Peter McNaughton, Dina Muktiarti, Satoshi Okada, Vu Van Quang, Amit Rawat, Monira Sarmin, Rajiva de Silva, Phub Tenzin, Lytheang Try, Youjia Zhong, Hsin-Hui Yu, Jane Chi Yan Wong, Yae-Jean Kim, Jaime S. Rosa Duque, Yu Lung Lau, Hirokazu Kanegane

**Affiliations:** 1 Department of Pediatrics and Developmental Biology, Graduate School of Medical and Dental Sciences, Institute of Science Tokyo, Tokyo, Japan; 2 Laboratory for Transcriptional Regulation, RIKEN Center for Integrative Medical Sciences, Yokohama, Japan; 3 Department of Rheumatology & Immunology Children’s Hospital of Chongqing Medical University, National Clinical Research Center for Child Health and Disorders, Ministry of Education Key Laboratory of Child Development and Disorders, Chongqing, China; 4 Advanced Center for Immunology and Rheumatology, Kathmandu, Nepal; 5 De La Salle Health Sciences Institute, Dasmarinas, Philippines; 6 Center of Excellence for Allergy and Clinical Immunology, Division of Allergy, Immunology, and Rheumatology, Department of Pediatrics, Faculty of Medicine, Chulalongkorn University, King Chulalongkorn Memorial Hospital, Bangkok, Thailand; 7 Department of Paediatrics, Faculty of Medicine and Health Sciences, Universiti Putra Malaysia, Serdang, Malaysia; 8 Non-Communicable Diseases Research Center, Alborz University of Medical Sciences, Karaj, Iran; 9 Department of Pediatrics, College of Medicine, The Catholic University of Korea, Seoul, Republic of Korea; 10 Queensland Paediatric Immunology and Allergy Service, Queensland Children’s Hospital, South Brisbane, Australia; 11 Department of Child Health, Faculty of Medicine Universitas Indonesia-Cipto Mangunkusumo Hospital, Jakarta, Indonesia; 12 Department of Pediatrics, Hiroshima University Graduate School of Biomedical and Health Sciences, Hiroshima, Japan; 13 Department of Pediatrics, Haiphong University of Medicine and Pharmacy, Haiphong, Vietnam; 14 Pediatric Allergy and Immunology Unit, Department of Pediatrics, Advanced Pediatrics Centre, Postgraduate Institute of Medical Education and Research, Chandigarh, India; 15 Nutrition Research Division, International Centre for Diarrhoeal Disease Research, Bangladesh (icddr,b), Dhaka, Bangladesh; 16 Department of Immunology, Medical Research Institute, Colombo, Sri Lanka; 17 Jigme Dorji Wangchuck National Referral Hospital, Thimphu, Bhutan; 18 Department of Pediatric Hemato-Immunology, National Pediatric Hospital, Phnom Penh, Cambodia; 19 Department of Paediatrics, Yong Loo Lin School of Medicine, National University of Singapore (NUS), Singapore, Singapore; 20 Division of Allergy, Immunology and Rheumatology, Department of Pediatrics, National Taiwan University Children’s Hospital, Taipei, Taiwan; 21 Division of Rheumatology and Clinical Immunology, Department of Medicine, Queen Mary Hospital, The University of Hong Kong, Hong Kong, China; 22 Department of Pediatrics, Samsung Medical Center, Sungkyunkwan University, School of Medicine, Seoul, Republic of Korea; 23 Department of Paediatrics and Adolescent Medicine, The University of Hong Kong, Hong Kong, China; 24 Department of Child Health and Development, Graduate School of Medical and Dental Sciences, Institute of Science Tokyo, Tokyo, Japan

## Abstract

Surveys of healthcare infrastructure for inborn errors of immunity across the Asia-Pacific region reveal significant diagnostic and therapeutic disparities. These data provide a framework for regional policy improvements and highlight the need for equitable resource distribution.

## Teaser

This questionnaire-based study provides insights into the current healthcare infrastructure used to diagnose and manage inborn errors of immunity across countries and regions involved in the Asia-Pacific Society for Immunodeficiencies.

**Figure 1.**
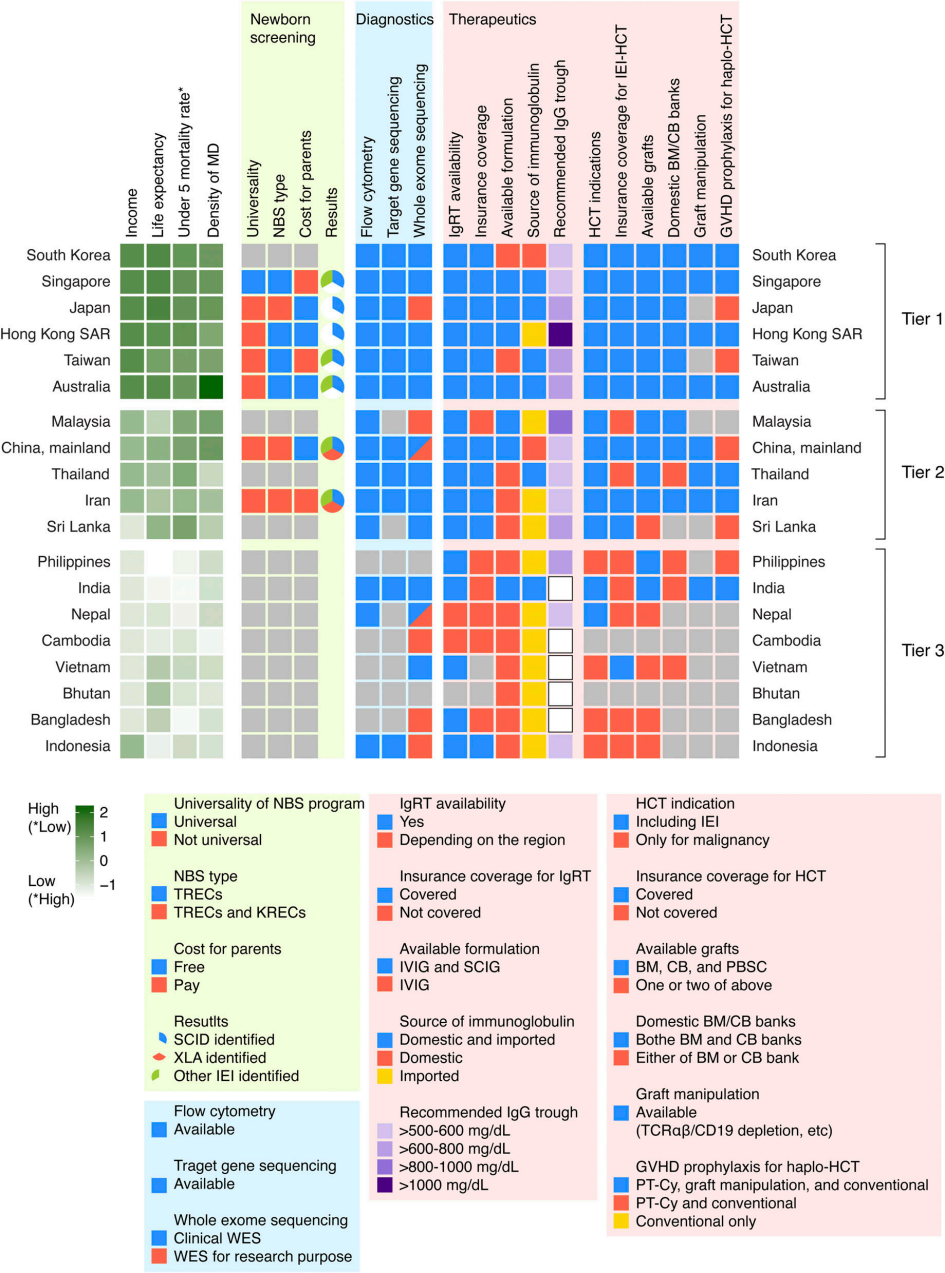
**Healthcare resources for patients with IEIs in the Asia-Pacific region.** The results of the questionnaire survey are summarized, with color codes and legends provided in the lower panel. BM, bone marrow; CB, cord blood; GVHD, graft-versus-host disease; HCT, hematopoietic cell transplantation; IEI, inborn error of immunity; IgG, immunoglobulin G; IgRT, immunoglobulin replacement therapy; IVIG, intravenous immunoglobulin; KRECs, kappa-deleting recombination excision circles; NBS, newborn screening; PBSC, peripheral blood stem cell; PT-Cy, posttransplantation cyclophosphamide; SCID, severe combined immunodeficiency; SCIG, subcutaneous immunoglobulin; TRECs, T-cell receptor excision circles; WES, whole-exome sequencing; XLA, X-linked agammaglobulinemia.

Basic and clinical research on inborn errors of immunity (IEIs) has significantly advanced our understanding of their pathogenesis and diverse clinical phenotypes, identifying nearly 500 distinct causative genes. However, healthcare resources for these patients, including diagnostic infrastructure and treatment options, vary widely across countries and regions (1). While previous studies have compared healthcare systems for IEI diagnosis and management in Southeast Asia (2), comprehensive knowledge of the situation across the Asia-Pacific region remains limited. In this study, we conducted a questionnaire-based survey through the Asia-Pacific Society for Immunodeficiencies (APSID) to assess the current state of key healthcare resources for IEI management in the Asia-Pacific region (results are summarized in Fig. 1).

Participating countries were categorized into three groups (Tiers 1–3) based on basic healthcare and economic indices. We selected the life expectancy at birth, the under-five mortality rate per 1,000 live births, and the density of medical doctors per 10,000 populations to assess general healthcare resources and status. Economic groups were defined according to the World Bank Income and Region report, classifying economies as low-income, lower-middle-income, upper-middle-income, and high-income, based on the gross national income per capita of ≦$1,145, $1,146–$4,515, $4,516–$14,005, and >$14,005, respectively. The variables were normalized by centering their mean values at 0. The values were normalized by subtracting the mean and dividing by the standard deviation, without applying any weighting to specific variables. Hierarchical clustering using the centroid method (via the stats package in R) was employed to group countries and regions. In brief, the clustering was based on the distances between cluster centroids, calculated as the mean values of each variable within a cluster. Countries and regions were then grouped according to their proximity to these centroids. Consequently, the hierarchical clustering identified three clusters (Tiers 1, 2, and 3) using the centroid method (Fig. 1). Tier 1 includes high-income economies with longer life expectancies, lower child mortality rates, and a higher density of medical doctors. Tier 2 includes upper-middle-income economies with moderate life expectancies, child mortality rates, and a moderate density of medical doctors. Tier 3 primarily consists of lower-middle-income economies with relatively short life expectancies, higher child mortality rates, and a lower density of medical doctors.

A newborn screening (NBS) program for IEI using kappa-deleting recombination excision circles (KRECs) and/or T-cell receptor excision circles (TRECs) has been introduced in Iran and mainland China in Tier 2 countries, as well as in all Tier 1 countries and regions, except South Korea (Fig. 1). Universal TREC screening is currently available only in Singapore, while other countries and regions either administer regional programs, are in the pilot stage, or offer optional testing. Japan and mainland China offer screening for KRECs, in addition to that for TRECs. Notably, adenosine deaminase (ADA)–deficient severe combined immunodeficiency (SCID) screening is offered at select hospitals in South Korea, successfully identifying the patients with ADA-SCID. In Singapore and Taiwan, parents bear the cost of screening. SCID and other IEIs have been identified in all countries and regions that have implemented TREC-based NBS. The cases of X-linked agammaglobulinemia (XLA) have been identified in countries and regions where KREC-based NBS has been introduced. None of the Tier 3 countries have initiated NBS programs for IEI.

Compared with NBS programs, flow cytometry and genetic testing for IEI are more widely available in APSID countries and regions (Fig. 1). Flow cytometry is accessible in all Tier 1 and Tier 2 countries and regions, and in three out of eight Tier 3 countries, excluding Bangladesh, Bhutan, Cambodia, the Philippines, and Vietnam. Similarly, target gene sequencing and whole-exome sequencing (WES) are available in the majority of countries and regions, with 11 and 17 out of 19, respectively, offering access to these diagnostic modalities. Importantly, WES is more widely available than target gene sequencing and is accessible in most APSID countries and regions, even where flow cytometry is not available.

Immunoglobulin replacement therapy (IgRT) is available in all surveyed countries and regions, except Bhutan (Fig. 1). However, among Tier 3 countries, only Indonesia covers this therapy through health insurance. Additionally, subcutaneous immunoglobulin (SCIG) therapy is available in all Tier 1 countries and regions, except South Korea and Taiwan; in two out of five Tier 2 countries; and in only India among the Tier 3 countries. The majority (7/8) of Tier 3 countries and three out of five Tier 2 countries rely on imported immunoglobulin products. The recommended trough levels did not differ across the tiers. Hematopoietic cell transplantation (HCT), a curative treatment for some IEIs, is available in all Tier 1 and Tier 2 countries and regions. However, four out of eight Tier 3 countries, specifically Bangladesh, the Philippines, Vietnam, and Indonesia, restrict HCT to the treatment of malignant diseases, and HCT is not available in Bhutan and Cambodia. In five out of six countries in Tier 3 where HCT is performed for IEI, excluding Vietnam, HCT is not covered by health insurance, similar to that in Malaysia and Thailand among the Tier 2 countries, making it financially challenging for patients. The availability of grafts and domestic bone marrow and/or cord blood banks is also limited in Tier 3 countries. T-cell receptor (TCR)-αβ/CD19 depletion is used as a graft manipulation technique for haploidentical HCT in eight countries and regions, including Australia, Hong Kong SAR, Singapore, South Korea, mainland China, Iran, India, and Thailand. Additionally, CD34 selection and CD45RA depletion are used in Australia, while CD3/CD45RA depletion is employed for haploidentical peripheral blood stem cell transplantation in Singapore. CD45RA and TCRαβ/CD19 depletion have been used for haploidentical HCT in Hong Kong SAR. Posttransplantation cyclophosphamide (PT-Cy) is widely used as a graft-versus-host disease prophylaxis in regions where haploidentical HCT is available.

Currently, universal TREC-based NBS is available only in Singapore. Expanding these programs to ensure that TREC-based NBS is universally accessible is a critical goal for these countries. In contrast to Tier 1 countries and regions, where TREC-based NBS is more widely available, three out of five Tier 2 and all of the Tier 3 countries have very limited access to NBS. The introduction of NBS in these regions will be crucial over the next decade, with growing evidence demonstrating that TREC-based NBS significantly improves clinical outcomes for patients with SCID (3). However, the rollout of NBS programs must be planned in parallel with the development of diagnostic and therapeutic infrastructure to support the care of identified patients. KREC-based NBS, which indicates B-cell generation capacity, has been successfully implemented in Japan and mainland China, where it has identified cases of XLA. Considering the inclusion of lower-income countries in APSID, evaluating the long-term impact and cost-effectiveness of screening for KRECs is essential before considering its widespread implementation in APSID regions.

Generally, genetic testing, including WES, is more accessible in APSID countries and regions than flow cytometry. This seemingly contradictory availability of WES in regions where flow cytometry is not accessible may stem from the logistical hurdles associated with transporting fresh samples for flow cytometry, compared with the relative ease of sample handling for genetic analysis, as well as the rapidly decreasing cost of WES. However, the functional validation of variants of uncertain significance, identified through these genetic tests, remains a key challenge, especially due to disparities in available resources. Similar to that for TREC-based NBS, it is critical to develop the necessary infrastructure for therapeutic systems to ensure that identified patients receive proper care. International collaborations in genetic diagnostics, such as the collaborations already underway in Nepal and India, will be increasingly important in overcoming resource limitations and ensuring accurate diagnoses across the region.

IgRT is a fundamental therapeutic intervention for patients with IEI, particularly those with primary antibody deficiencies and combined immunodeficiencies. The therapy is widely available in APSID countries and regions, but is not universally accessible. Ensuring the introduction and accessibility of IgRT is a critical issue in Tier 3 countries and regions, where availability is limited, and healthcare insurance coverage is often inadequate. Additionally, the reliance on overseas supplies of intravenous immunoglobulin (IVIG) or SCIG products poses challenges. The growing demand for IgRT, coupled with the disproportionate dependence on limited production sources, has led to global shortages and an unreliable supply of immunoglobulin products (4). Therefore, establishing a stable and sustainable production capacity within the region has become a critical priority. SCIG, which is widely available in resource-rich countries, offers the advantages of home administration, reducing the need for frequent hospital visits, and is associated with economic benefits compared with IVIG therapy (5). Thus, SCIG could be a viable solution in regions with limited healthcare resources. The recommended IgG trough levels for IgRT vary by region and are not consistently higher in Tier 1 countries and regions. Although higher trough levels are associated with better clinical outcomes, establishing cost-effective trough levels is particularly important in resource-limited settings.

The availability of HCT for IEI is limited in Tier 3 countries. Graft manipulation is available only in a few countries and regions, including Australia, Hong Kong SAR, mainland China, Iran, Singapore, South Korea, and Thailand, and has been used for haploidentical transplants. PT-Cy is also widely used in countries where haploidentical transplants are performed: its cost-effectiveness could offer significant benefits for many APSID countries.

A limitation of this study is its reliance on a questionnaire survey, which may have led to discrepancies between reported answers and actual practices and infrastructure. Additionally, this study is limited to countries and regions with active APSID members and does not cover the entire Asia-Pacific region. Although the results are intended to reflect nationwide practices, there may still be region-specific differences in healthcare infrastructure.

This questionnaire-based study provides insights into the current healthcare infrastructure for diagnosing and managing IEI in APSID countries and regions. The findings of this study highlight the limited implementation of TREC-based NBS programs, while revealing a surprisingly high availability of WES services. However, essential treatments for IEI, such as IgRT and HCT, remain inaccessible in many lower-middle-income countries. Addressing these gaps will be a critical challenge in the coming decades. Longitudinal studies are required for monitoring improvements in IEI healthcare infrastructure over time, with outcome-based research being essential for assessing the effectiveness and cost efficiency of management systems across different economic contexts. Given the varying levels of healthcare resources available for the management of IEI, the standardization of care, tailored to different resource settings, could be effectively addressed through the development of clinical guidelines that take these disparities into account. Such an initiative should be advocated and led by APSID. Furthermore, considering the current landscape in which international collaboration is already active, particularly in the field of genetic diagnosis, the centralization of genetic and immunologic diagnostic services within the Asia-Pacific region appears both feasible and highly beneficial. International collaboration in diagnosis and management, along with efforts to bridge these gaps, will be crucial for the future of IEI healthcare.
